# Supplementary material for: Disease-Specific Quality Indicators for Outpatient Antibiotic Prescribing for Respiratory Infections (ESAC Quality Indicators) Applied to Point Prevalence Audit Surveys in General Practices in 13 European Countries
Source: Antibiotics (Basel). 2023 Mar 14;12(3):572. doi: 10.3390/antibiotics12030572 (PMC10044809; doi:10.3390/antibiotics12030572)
Supplement: Supplementary file 1 [file antibiotics-12-00572-s001.zip › antibiotics-2213577-supplementary.pdf]

Table S1. Overview of all ESAC QI for PPAS 1 and PPAS 4, overall and by country, including the acceptable range.

For indicators which were calculated twice, due to the adjustments made to the penicillin categories, numbers are highlighted red only for the calculated percentage which combined all penicillins into one group.

All:

Indicator ID	Short Indicator Description, with modifications for this study	Acceptable Range (%)	PPAS 1(n)	PPAS 1 (%)	PPAS 4 (n)	PPAS 4 (%)
ESAC 1A	Percentage of patients aged between 18-75 with acute bronchitis/bronchiolitis prescribed antibacterials	0-30	197	45.2	64	56.6
ESAC1B	= 1a receiving the recommended antibacterials: broad spectrum penicillin[a] or tetracycline	80-100	78	39.6	27	42.2
ESAC1B	=1a receiving the recommended antibacterials: any pencillin[b] or tetracycline	80-100	84	42.6	28	43.8
ESAC1C	= 1a receiving quinolones	0-5	7	3.6	1	1.6
ESAC2A	Percentage of patients older than 1 year with acute upper respiratory infection, cold, or sinusitis, prescribed antibacterials	0-20	56	12.8	77	9.6
ESAC2B	=2a receiving the recommended antibacterials: narrow spectrum penicillin[a]	80-100	2	3.6	4	5.2
ESAC2B	=2a receiving the recommended antibacterials: any pencillin[b]	80-100	37	66.1	39	50.6
ESAC2C	=2a receiving quinolones	0-5	2	3.6	5	6.5
ESAC4A	Percentage of patients older than 1 year with acute tonsillitis prescribed antibacterials	0-20	268	69.3	80	76.2
ESAC4B	=4a receiving the recommended antibacterials: narrow spectrum penicillin[a]	80-100	112	41.8	42	52.5
ESAC4B	=4a receiving the recommended antibacterials: any pencillin[b]	80-100	157	58.6	53	66.3
ESAC4C	=4a receiving quinolones	0-5	2	0.7	0	0
ESAC5A	Percentage of patients older than 18 years with sinusitis, upper respiratory tract infection, or cold prescribed an antibacterial	0-20	45	15.0	57	10.2
ESAC5B	=5a receiving the recommended antibacterials: any penicillin[b]	80-100	30	66.7	25	43.9
ESAC5C	=5a receiving quinolones	0-5	1	2.2	4	7

ESAC7A	Percentage of patients aged between 18 and 65 prescribed antibacterials	90-100	102	78.5	52	86.7
ESAC7B	=7a receiving the recommended antibacterials: broad spectrum penicillin[a] or tetracycline	80-100	42	41.2	28	53.8
ESAC7B	=7a receiving the recommended antibacterials: any penicillin[b] or tetracycline	80-100	54	52.9	31	59.6
ESAC7C	=7a receiving quinolones	0-5	8	7.8	2	3.8

[a]Broad spectrum penicillin = J01CA: Extended spectrum penicillins; Narrow spectrum penicillin = J01CE, beta-lactamase sensitive penicillins

[b]Broad spectrum and narrow penicillins combined into one category applied to both J01CA and J01CE

Belgium:

		Acceptable Range (%)	PPAS 1(n)	PPAS 1 (%)	PPAS 4 (n)	PPAS 4 (%)
ESAC 1A	Percentage of patients aged between 18-75 with acute bronchitis/bronchiolitis prescribed antibacterials	0-30	9	31	NA	NA
ESAC1B[a]	= 1a receiving the recommended antibacterials: broad spectrum penicillin or tetracycline	80-100	6	67	NA	NA
ESAC1B[b]	=1a receiving the recommended antibacterials: any penicillin or tetracycline	80-100	6	67	NA	NA
ESAC1C	= 1a receiving quinolones	0-5	0	0	NA	NA
ESAC2A	Percentage of patients older than 1 year with acute upper respiratory infection, cold, or sinusitis, prescribed antibacterials	0-20	5	7	1	2
ESAC2B[a]	=2a receiving the recommended antibacterials: narrow spectrum penicillin	80-100	0	0	0	0
ESAC2B[b]	=2a receiving the recommended antibacterials: any penicillin	80-100	4	80	1	100
ESAC2C	=2a receiving quinolones	0-5	0	0	0	0
ESAC4A	Percentage of patients older than 1 year with acute tonsillitis prescribed antibacterials	0-20	16	36	2	67

ESAC4B[a]	=4a receiving the recommended antibacterials: narrow spectrum penicillin	80-100	0	0	0	0
ESAC4B[b]	=4a receiving the recommended antibacterials: any penicillin	80-100	10	63	2	100
ESAC4C	=4a receiving quinolones	0-5	0	0	0	0
ESAC5A	Percentage of patients older than 18 years with sinusitis, upper respiratory tract infection, or cold prescribed an antibacterial	0-20	4	8	1	2
ESAC5B[b]	=5a receiving the recommended antibacterials: any penicillin	80-100	3	75	1	100
ESAC5C	=5a receiving quinolones	0-5	0	0	0	0
ESAC7A	Percentage of patients aged between 18 and 65 prescribed antibacterials	90-100	9	82	1	100
ESAC7B[a]	=7a receiving the recommended antibacterials: broad spectrum penicillin or tetracycline	80-100	7	78	0	0
ESAC7B[b]	=7a receiving the recommended antibacterials: any penicillin or tetracycline	80-100	7	78	0	0
ESAC7C	=7a receiving quinolones	0-5	0	0	0	0

[a]Broad spectrum penicillin = J01CA: Extended spectrum penicillins; Narrow spectrum penicillin = J01CE, beta-lactamase sensitive penicillins

[b]Broad spectrum and narrow penicillins combined into one category applied to both J01CA and J01CE

Croatia:

		Acceptable Range (%)	PPAS 1(n)	PPAS 1 (%)	PPAS 4 (n)	PPAS 4 (%)
ESAC 1A	Percentage of patients aged between 18-75 with acute bronchitis/bronchiolitis prescribed antibacterials	0-30	10	37	1	33
ESAC1B[a]	= 1a receiving the recommended antibacterials: broad spectrum penicillin or tetracycline	80-100	1	10	0	0

ESAC1B[b]	=1a receiving the recommended antibacterials: any penicillin or tetracycline	80-100	1	10	0	0
ESAC1C	= 1a receiving quinolones	0-5	0	0	0	0
ESAC2A	Percentage of patients older than 1 year with acute upper respiratory infection, cold, or sinusitis, prescribed antibacterials	0-20	4	44	2	9
ESAC2B[a]	=2a receiving the recommended antibacterials: narrow spectrum penicillin	80-100	0	0	0	0
ESAC2B[b]	=2a receiving the recommended antibacterials: any penicillin	80-100	1	25	0	0
ESAC2C	=2a receiving quinolones	0-5	0	0	0	0
ESAC4A	Percentage of patients older than 1 year with acute tonsillitis prescribed antibacterials	0-20	6	40	1	25
ESAC4B[a]	=4a receiving the recommended antibacterials: narrow spectrum penicillin	80-100	5	83	1	100
ESAC4B[b]	=4a receiving the recommended antibacterials: any penicillin	80-100	5	83	1	100
ESAC4C	=4a receiving quinolones	0-5	0	0	0	0
ESAC5A	Percentage of patients older than 18 years with sinusitis, upper respiratory tract infection, or cold prescribed an antibacterial	0-20	3	60	2	13
ESAC5B[b]	=5a receiving the recommended antibacterials: any penicillin	80-100	0	0	0	0
ESAC5C	=5a receiving quinolones	0-5	0	0	0	0
ESAC7A	Percentage of patients aged between 18 and 65 prescribed antibacterials	90-100	8	89	2	100
ESAC7B[a]	=7a receiving the recommended antibacterials: broad spectrum penicillin or tetracycline	80-100	0	0	0	0
ESAC7B[b]	=7a receiving the recommended antibacterials: any penicillin or tetracycline	80-100	0	0	0	0

ESAC7C	=7a receiving quinolones	0-5	0	0	0	0

[a]Broad spectrum penicillin = J01CA: Extended spectrum penicillins; Narrow spectrum penicillin = J01CE, beta-lactamase sensitive penicillins

[b]Broad spectrum and narrow penicillins combined into one category applied to both J01CA and J01CE

Denmark:

		Acceptable Range (%)	PPAS 1(n)	PPAS 1 (%)	PPAS 4 (n)	PPAS 4 (%)
ESAC 1A	Percentage of patients aged between 18-75 with acute bronchitis/bronchiolitis prescribed antibacterials	0-30	7	14	0	0
ESAC1B[a]	= 1a receiving the recommended antibacterials: broad spectrum penicillin or tetracycline	80-100	0	0	NA	NA
ESAC1B[b]	=1a receiving the recommended antibacterials: any penicillin or tetracycline	80-100	5	71	NA	NA
ESAC1C	= 1a receiving quinolones	0-5	0	0	NA	NA
ESAC2A	Percentage of patients older than 1 year with acute upper respiratory infection, cold, or sinusitis, prescribed antibacterials	0-20	2	7	0	0
ESAC2B[a]	=2a receiving the recommended antibacterials: narrow spectrum penicillin	80-100	2	100	NA	NA
ESAC2B[b]	=2a receiving the recommended antibacterials: any penicillin	80-100	2	100	NA	NA
ESAC2C	=2a receiving quinolones	0-5	0	0	NA	NA
ESAC4A	Percentage of patients older than 1 year with acute tonsillitis prescribed antibacterials	0-20	32	84	13	87
ESAC4B[a]	=4a receiving the recommended antibacterials: narrow spectrum penicillin	80-100	31	97	10	77
ESAC4B[b]	=4a receiving the recommended antibacterials: any penicillin	80-100	31	97	11	85

ESAC4C	=4a receiving quinolones	0-5	0	0	0	0
ESAC5A	Percentage of patients older than 18 years with sinusitis, upper respiratory tract infection, or cold prescribed an antibacterial	0-20	2	10	0	0
ESAC5B[b]	=5a receiving the recommended antibacterials: any penicillin	80-100	2	100	NA	NA
ESAC5C	=5a receiving quinolones	0-5	0	0	NA	NA
ESAC7A	Percentage of patients aged between 18 and 65 prescribed antibacterials	90-100	16	59	3	50
ESAC7B[a]	=7a receiving the recommended antibacterials: broad spectrum penicillina or tetracycline	80-100	0	0	0	0
ESAC7B[b]	=7a receiving the recommended antibacterials: any penicillin or tetracycline	80-100	12	75	3	100
ESAC7C	=7a receiving quinolones	0-5	0	0	0	0

[a]Broad spectrum penicillin = J01CA: Extended spectrum penicillins; Narrow spectrum penicillin = J01CE, beta-lactamase sensitive penicillins
[b]Broad and narrow penicillins combined into one category applied to both J01CA and J01CE

Georgia:

		Acceptable Range (%)	PPAS 1(n)	PPAS 1 (%)	PPAS 4 (n)	PPAS 4 (%)
ESAC 1A	Percentage of patients aged between 18-75 with acute bronchitis/bronchiolitis prescribed antibacterials	0-30	22	42	10	63
ESAC1B[a]	= 1a receiving the recommended antibacterials: broad spectrum penicillin or tetracycline	80-100	1	5	2	20
ESAC1B[b]	=1a receiving the recommended antibacterials: any penicillin or tetracycline	80-100	1	5	2	20
ESAC1C	= 1a receiving quinolones	0-5	1	5	0	0

ESAC2A	Percentage of patients older than 1 year with acute upper respiratory infection, cold, or sinusitis, prescribed antibacterials	0-20	1	9	4	7
ESAC2B[a]	=2a receiving the recommended antibacterials: narrow spectrum penicillin	80-100	0	0	0	0
ESAC2B[b]	=2a receiving the recommended antibacterials: any penicillin	80-100	1	100	0	0
ESAC2C	=2a receiving quinolones	0-5	0	0	0	0
ESAC4A	Percentage of patients older than 1 year with acute tonsillitis prescribed antibacterials	0-20	11	85	4	100
ESAC4B[a]	=4a receiving the recommended antibacterials: narrow spectrum penicillin	80-100	0	0	0	0
ESAC4B[b]	=4a receiving the recommended antibacterials: any penicillin	80-100	2	18	0	0
ESAC4C	=4a receiving quinolones	0-5	0	0	0	0
ESAC5A	Percentage of patients older than 18 years with sinusitis, upper respiratory tract infection, or cold prescribed an antibacterial	0-20	1	14	3	7
ESAC5B[b]	=5a receiving the recommended antibacterials: any penicillin	80-100	1	100	0	0
ESAC5C	=5a receiving quinolones	0-5	0	0	0	0
ESAC7A	Percentage of patients aged between 18 and 65 prescribed antibacterials	90-100	3	75	9	100
ESAC7B[a]	=7a receiving the recommended antibacterials: broad spectrum penicillin or tetracycline	80-100	0	0	0	0
ESAC7B[b]	=7a receiving the recommended antibacterials: any penicillin or tetracycline	80-100	0	0	0	0
ESAC7C	=7a receiving quinolones	0-5	0	0	0	0

[a]Broad spectrum penicillin = J01CA: Extended spectrum penicillins; Narrow spectrum penicillin = J01CE, beta-lactamase sensitive penicillins

[b]Broad spectrum and narrow penicillins combined into one category applied to both J01CA and J01CE

Germany:

		Acceptable Range (%)	PPAS 1(n)	PPAS 1 (%)	PPAS 4 (n)	PPAS 4 (%)
ESAC 1A	Percentage of patients aged between 18-75 with acute bronchitis/bronchiolitis prescribed antibacterials	0-30	10	100	6	55
ESAC1 B[a]	= 1a receiving the recommended antibacterials: broad spectrum penicillin or tetracycline	80-100	6	60	2	33
ESAC1 B[b]	=1a receiving the recommended antibacterials: any penicillin or tetracycline	80-100	6	60	2	33
ESAC1 C	= 1a receiving quinolones	0-5	0	0	0	0
ESAC2 A	Percentage of patients older than 1 year with acute upper respiratory infection, cold, or sinusitis, prescribed antibacterials	0-20	6	10	12	10
ESAC2 B[a]	=2a receiving the recommended antibacterials: narrow spectrum penicillin	80-100	0	0	0	0
ESAC2 B[b]	=2a receiving the recommended antibacterials: any penicillin	80-100	5	83	7	58
ESAC2 C	=2a receiving quinolones	0-5	0	0	2	17
ESAC4 A	Percentage of patients older than 1 year with acute tonsillitis prescribed antibacterials	0-20	8	67	3	75
ESAC4 B[a]	=4a receiving the recommended antibacterials: narrow spectrum penicillin	80-100	7	88	1	33
ESAC4 B[b]	=4a receiving the recommended antibacterials: any penicillin	80-100	8	100	1	33
ESAC4 C	=4a receiving quinolones	0-5	0	0	0	0
ESAC5 A	Percentage of patients older than 18 years with sinusitis, upper respiratory tract	0-20	6	11	12	10

	infection, or cold prescribed an antibacterial					
ESAC5 B[b]	=5a receiving the recommended antibacterials: any penicillin	80-100	5	84	7	58
ESAC5 C	=5a receiving quinolones	0-5	0	0	2	17
ESAC7 A	Percentage of patients aged between 18 and 65 prescribed antibacterials	90-100	4	100	6	60
ESAC7 B[a]	=7a receiving the recommended antibacterials: broad spectrum penicillin or tetracycline	80-100	2	50	5	83
ESAC7 B[b]	=7a receiving the recommended antibacterials: any penicillin or tetracycline	80-100	2	50	5	83
ESAC7 C	=7a receiving quinolones	0-5	0	0	0	0

[a]Broad spectrum penicillin = J01CA: Extended spectrum penicillins; Narrow spectrum penicillin = J01CE, beta-lactamase sensitive penicillins
[b]Broad spectrum and narrow penicillins combined into one category applied to both J01CA and J01CE

Greece:

		Acceptable Range (%)	PPAS 1(n)	PPAS 1 (%)	PPAS 4 (n)	PPAS 4 (%)
ESAC 1A	Percentage of patients aged between 18-75 with acute bronchitis/bronchiolitis prescribed antibacterials	0-30	30	75	11	58
ESAC1 B[a]	= 1a receiving the recommended antibacterials: broad spectrum penicillin or tetracycline	80-100	0	0	0	0
ESAC1 B[b]	=1a receiving the recommended antibacterials: any penicillin or tetracycline	80-100	0	0	0	0
ESAC1 C	= 1a receiving quinolones	0-5	4	13	1	9

ESAC2 A	Percentage of patients older than 1 year with acute upper respiratory infection, cold, or sinusitis, prescribed antibacterials	0-20	NA	NA	5	26
ESAC2 B[a]	=2a receiving the recommended antibacterials: narrow spectrum penicillin	80-100	NA	NA	0	0
ESAC2 B[b]	=2a receiving the recommended antibacterials: any penicilli[n]	80-100	NA	NA	0	0
ESAC2 C	=2a receiving quinolones	0-5	NA	NA	2	40
ESAC4 A	Percentage of patients older than 1 year with acute tonsillitis prescribed antibacterials	0-20	8	73	5	100
ESAC4 B[a]	=4a receiving the recommended antibacterials: narrow spectrum penicillin	80-100	0	0	0	0
ESAC4 B[b]	=4a receiving the recommended antibacterials: any penicillin	80-100	0	0	0	0
ESAC4 C	=4a receiving quinolones	0-5	0	0	0	0
ESAC5 A	Percentage of patients older than 18 years with sinusitis, upper respiratory tract infection, or cold prescribed an antibacterial	0-20	NA	NA	5	28
ESAC5 B[b]	=5a receiving the recommended antibacterials: any penicillin	80-100	NA	NA	0	0
ESAC5 C	=5a receiving quinolones	0-5	NA	NA	2	40
ESAC7 A	Percentage of patients aged between 18 and 65 prescribed antibacterials	90-100	6	86	3	100
ESAC7 B[a]	=7a receiving the recommended antibacterials: broad spectrum penicillin or tetracycline	80-100	0	0	0	0
ESAC7 B[b]	=7a receiving the recommended antibacterials: any penicillin or tetracycline	80-100	0	0	0	0
ESAC7 C	=7a receiving quinolones	0-5	4	67	1	33

[a]Broad spectrum penicillin = J01CA: Extended spectrum penicillins; Narrow spectrum penicillin = J01CE, beta-lactamase sensitive penicillins
[b]Broad spectrum and narrow penicillins combined into one category applied to both J01CA and J01CE

Ireland:

		Acceptable Range (%)	PPAS 1(n)	PPAS 1 (%)	PPAS 4 (n)	PPAS 4 (%)
ESAC 1A	Percentage of patients aged between 18-75 with acute bronchitis/bronchiolitis prescribed antibacterials	0-30	26	74	4	67
ESAC1 B[a]	= 1a receiving the recommended antibacterials: broad spectrum penicillin or tetracycline	80-100	16	62	3	75
ESAC1 B[b]	=1a receiving the recommended antibacterials: any penicillin or tetracycline	80-100	16	62	3	75
ESAC1 C	= 1a receiving quinolones	0-5	0	0	0	0
ESAC2 A	Percentage of patients older than 1 year with acute upper respiratory infection, cold, or sinusitis, prescribed antibacterials	0-20	24	49	22	40
ESAC2 B[a]	=2a receiving the recommended antibacterials: narrow spectrum penicillin	80-100	0	0	3	14
ESAC2 B[b]	=2a receiving the recommended antibacterials: any penicillin	80-100	16	67	12	55
ESAC2 C	=2a receiving quinolones	0-5	1	4	0	0
ESAC4 A	Percentage of patients older than 1 year with acute tonsillitis prescribed antibacterials	0-20	21	81	10	83
ESAC4 B[a]	=4a receiving the recommended antibacterials: narrow spectrum penicillin	80-100	7	33	6	60
ESAC4 B[b]	=4a receiving the recommended antibacterials: any penicillin	80-100	11	52	8	80

ESAC4 C	=4a receiving quinolones	0-5	0	0	0	0
ESAC5 A	Percentage of patients older than 18 years with sinusitis, upper respiratory tract infection, or cold prescribed an antibacterial	0-20	19	48	14	47
ESAC5 B[b]	=5a receiving the recommended antibacterials: any penicillin	80-100	13	68	6	43
ESAC5 C	=5a receiving quinolones	0-5	0	0	0	0
ESAC7 A	Percentage of patients aged between 18 and 65 prescribed antibacterials	90-100	9	82	3	100
ESAC7 B[a]	=7a receiving the recommended antibacterials: broad spectrum penicillin or tetracycline	80-100	5	56	3	100
ESAC7 B[b]	=7a receiving the recommended antibacterials: any penicillin or tetracycline	80-100	5	56	3	100
ESAC7 C	=7a receiving quinolones	0-5	0	0	0	0

[a]Broad spectrum penicillin = J01CA: Extended spectrum penicillins; Narrow spectrum penicillin = J01CE, beta-lactamase sensitive penicillins
[b]Broad spectrum and narrow penicillins combined into one category applied to both J01CA and J01CE

Moldova:

		Acceptable Range (%)	PPAS 1(n)	PPAS 1 (%)	PPAS 4 (n)	PPAS 4 (%)
ESAC 1A	Percentage of patients aged between 18-75 with acute bronchitis/bronchiolitis prescribed antibacterials	0-30	13	87	NA	NA
ESAC1 B[a]	= 1a receiving the recommended antibacterials: broad spectrum penicillin or tetracycline	80-100	3	23	NA	NA
ESAC1 B[b]	=1a receiving the recommended	80-100	3	23	NA	NA

	antibacterials: any penicillin or tetracycline					
ESAC1 C	= 1a receiving quinolones	0-5	0	0	NA	NA
ESAC2 A	Percentage of patients older than 1 year with acute upper respiratory infection, cold, or sinusitis, prescribed antibacterials	0-20	2	3	0	0
ESAC2 B[a]	=2a receiving the recommended antibacterials: narrow spectrum penicillin	80-100	0	0	NA	NA
ESAC2 B[b]	=2a receiving the recommended antibacterials: any penicillin	80-100	0	0	NA	NA
ESAC2 C	=2a receiving quinolones	0-5	0	0	NA	NA
ESAC4 A	Percentage of patients older than 1 year with acute tonsillitis prescribed antibacterials	0-20	45	96	0	0
ESAC4 B[a]	=4a receiving the recommended antibacterials: narrow spectrum penicillin	80-100	0	0	NA	NA
ESAC4 B[b]	=4a receiving the recommended antibacterials: any penicillin	80-100	14	31	NA	NA
ESAC4 C	=4a receiving quinolones	0-5	0	0	0	0
ESAC5 A	Percentage of patients older than 18 years with sinusitis, upper respiratory tract infection, or cold prescribed an antibacterial	0-20	1	4	0	0
ESAC5 B[b]	=5a receiving the recommended antibacterials: any penicillin	80-100	0	0	NA	NA
ESAC5 C	=5a receiving quinolones	0-5	0	0	NA	NA
ESAC7 A	Percentage of patients aged between 18 and 65 prescribed antibacterials	90-100	1	100	NA	NA
ESAC7 B[a]	=7a receiving the recommended antibacterials: broad spectrum penicillin or tetracycline	80-100	0	0	NA	NA

ESAC7 B[b]	=7a receiving the recommended antibacterials: any penicillin or tetracycline	80-100	0	0	NA	NA
ESAC7 C	=7a receiving quinolones	0-5	0	0	0	0

[a]Broad spectrum penicillin = J01CA: Extended spectrum penicillins; Narrow spectrum penicillin = J01CE, beta-lactamase sensitive penicillins

[b]Broad spectrum and narrow penicillins combined into one category applied to both J01CA and J01CE

Netherlands:

		Acceptable Range (%)	PPAS 1(n)	PPAS 1 (%)	PPAS 4 (n)	PPAS 4 (%)
ESAC 1A	Percentage of patients aged between 18-75 with acute bronchitis/bronchiolitis prescribed antibacterials	0-30	13	42	3	50
ESAC1 B[a]	= 1a receiving the recommended antibacterials: broad spectrum penicillin or tetracycline	80-100	12	92	2	67
ESAC1 B[b]	=1a receiving the recommended antibacterials: any penicillin or tetracycline	80-100	12	92	2	67
ESAC1 C	= 1a receiving quinolones	0-5	0	0	0	0
ESAC2 A	Percentage of patients older than 1 year with acute upper respiratory infection, cold, or sinusitis, prescribed antibacterials	0-20	8	14	11	11
ESAC2 B[a]	=2a receiving the recommended antibacterials: narrow spectrum penicillin	80-100	0	0	0	0
ESAC2 B[b]	=2a receiving the recommended antibacterials: any penicillin	80-100	7	88	9	82
ESAC2 C	=2a receiving quinolones	0-5	0	0	0	0
ESAC4 A	Percentage of patients older than 1 year with acute tonsillitis prescribed antibacterials	0-20	7	42	4	40

ESAC4 B[a]	=4a receiving the recommended antibacterials: narrow spectrum penicillin	80-100	6	86	1	25
ESAC4 B[b]	=4a receiving the recommended antibacterials: any penicillin	80-100	7	100	1	25
ESAC4 C	=4a receiving quinolones	0-5	0	0	0	0
ESAC5 A	Percentage of patients older than 18 years with sinusitis, upper respiratory tract infection, or cold prescribed an antibacterial	0-20	5	11	6	11
ESAC5 B[b]	=5a receiving the recommended antibacterials: any penicillin	80-100	5	100	4	67
ESAC5 C	=5a receiving quinolones	0-5	0	0	0	0
ESAC7 A	Percentage of patients aged between 18 and 65 prescribed antibacterials	90-100	26	84	5	83
ESAC7 B[a]	=7a receiving the recommended antibacterials: broad spectrum penicillin or tetracycline	80-100	24	92	4	80
ESAC7 B[b]	=7a receiving the recommended antibacterials: any penicillin or tetracycline	80-100	24	92	4	80
ESAC7 C	=7a receiving quinolones	0-5	1	4	0	0

[a]Broad spectrum penicillin = J01CA: Extended spectrum penicillins; Narrow spectrum penicillin = J01CE, beta-lactamase sensitive penicillins

[b]Broad spectrum and narrow penicillins combined into one category applied to both J01CA and J01CE

Poland:

		Acceptable Range (%)	PPAS 1(n)	PPAS 1 (%)	PPAS 4 (n)	PPAS 4 (%)
ESAC 1A	Percentage of patients aged between 18-75 with acute bronchitis/bronchiolitis prescribed antibacterials	0-30	8	67	4	67
ESAC1 B[a]	= 1a receiving the recommended antibacterials: broad	80-100	1	13	0	0

	spectrum penicillin or tetracycline					
ESAC1 B[b]	=1a receiving the recommended antibacterials: any penicillin or tetracycline	80-100	1	13	0	0
ESAC1 C	= 1a receiving quinolones	0-5	0	0	0	0
ESAC2 A	Percentage of patients older than 1 year with acute upper respiratory infection, cold, or sinusitis, prescribed antibacterials	0-20	0	0	6	7
ESAC2 B[a]	=2a receiving the recommended antibacterials: narrow spectrum penicillin	80-100	NA	NA	1	17
ESAC2 B[b]	=2a receiving the recommended antibacterials: any penicillin	80-100	NA	NA	3	50
ESAC2 C	=2a receiving quinolones	0-5	NA	NA	1	17
ESAC4 A	Percentage of patients older than 1 year with acute tonsillitis prescribed antibacterials	0-20	10	71	2	100
ESAC4 B[a]	=4a receiving the recommended antibacterials: narrow spectrum penicillin	80-100	5	50	1	50
ESAC4 B[b]	=4a receiving the recommended antibacterials: any penicillin	80-100	5	50	2	100
ESAC4 C	=4a receiving quinolones	0-5	0	0	0	0
ESAC5 A	Percentage of patients older than 18 years with sinusitis, upper respiratory tract infection, or cold prescribed an antibacterial	0-20	0	0	2	5
ESAC5 B[b]	=5a receiving the recommended antibacterials: any penicillin	80-100	NA	NA	1	50
ESAC5 C	=5a receiving quinolones	0-5	NA	NA	0	0
ESAC7 A	Percentage of patients aged between 18 and 65 prescribed antibacterials	90-100	6	100	5	100

ESAC7 B[a]	=7a receiving the recommended antibacterials: broad spectrum penicillin or tetracycline	80-100	0	0	3	60
ESAC7 B[b]	=7a receiving the recommended antibacterials: any penicillin or tetracycline	80-100	0	0	3	60
ESAC7 C	=7a receiving quinolones	0-5	0	0	1	20

[a]Broad spectrum penicillin = J01CA: Extended spectrum penicillins; Narrow spectrum penicillin = J01CE, beta-lactamase sensitive penicillins
[b]Broad spectrum and narrow penicillins combined into one category applied to both J01CA and J01CE

Romania:

		Acceptable Range (%)	PPAS 1(n)	PPAS 1 (%)	PPAS 4 (n)	PPAS 4 (%)
ESAC 1A	Percentage of patients aged between 18-75 with acute bronchitis/bronchiolitis prescribed antibacterials	0-30	12	43	4	100
ESAC1B[a]	= 1a receiving the recommended antibacterials: broad spectrum penicillin or tetracycline	80-100	0	0	0	0
ESAC1B[b]	=1a receiving the recommended antibacterials: any penicillin or tetracycline	80-100	0	0	0	0
ESAC1C	= 1a receiving quinolones	0-5	2	17	0	0
ESAC2A	Percentage of patients older than 1 year with acute upper respiratory infection, cold, or sinusitis, prescribed antibacterials	0-20	2	13	0	0
ESAC2B[a]	=2a receiving the recommended antibacterials: narrow spectrum penicillin	80-100	0	0	NA	NA
ESAC2B[b]	=2a receiving the recommended antibacterials: any penicillin	80-100	0	0	NA	NA
ESAC2C	=2a receiving quinolones	0-5	1	50	NA	NA

ESAC4A	Percentage of patients older than 1 year with acute tonsillitis prescribed antibacterials	0-20	38	61	6	60
ESAC4B[a]	=4a receiving the recommended antibacterials: narrow spectrum penicillin	80-100	4	11	1	17
ESAC4B[b]	=4a receiving the recommended antibacterials: any penicillin	80-100	7	18	1	17
ESAC4C	=4a receiving quinolones	0-5	2	5	0	0
ESAC5A	Percentage of patients older than 18 years with sinusitis, upper respiratory tract infection, or cold prescribed an antibacterial	0-20	2	15	0	0
ESAC5B[b]	=5a receiving the recommended antibacterials: any penicillin	80-100	0	0	NA	NA
ESAC5C	=5a receiving quinolones	0-5	1	50	NA	NA
ESAC7A	Percentage of patients aged between 18 and 65 prescribed antibacterials	90-100	4	67	2	100
ESAC7B[a]	=7a receiving the recommended antibacterials: broad spectrum penicillin or tetracycline	80-100	0	0	0	0
ESAC7B[b]	=7a receiving the recommended antibacterials: any penicillin or tetracycline	80-100	0	0	0	0
ESAC7C	=7a receiving quinolones	0-5	0	0	0	0

[a]Broad spectrum penicillin = J01CA: Extended spectrum penicillins; Narrow spectrum penicillin = J01CE, beta-lactamase sensitive penicillins

[b]Broad spectrum and narrow penicillins combined into one category applied to both J01CA and J01CE

Spain:

		Acceptable Range (%)	PPAS 1(n)	PPAS 1 (%)	PPAS 4 (n)	PPAS 4 (%)
ESAC 1A	Percentage of patients aged between 18-75 with acute bronchitis/bronchiolitis prescribed antibacterials	0-30	9	13	0	0

ESAC1 B[a]	= 1a receiving the recommended antibacterials: broad spectrum penicillin or tetracycline	80-100	7	78	NA	NA
ESAC1 B[b]	=1a receiving the recommended antibacterials: any penicillin or tetracycline	80-100	7	78	NA	NA
ESAC1 C	= 1a receiving quinolones	0-5	0	0	NA	NA
ESAC2 A	Percentage of patients older than 1 year with acute upper respiratory infection, cold, or sinusitis, prescribed antibacterials	0-20	2	15	5	8
ESAC2 B[a]	=2a receiving the recommended antibacterials: narrow spectrum penicillin	80-100	0	0	0	0
ESAC2 B[b]	=2a receiving the recommended antibacterials: any penicillin	80-100	1	50	3	60
ESAC2 C	=2a receiving quinolones	0-5	0	0	0	0
ESAC4 A	Percentage of patients older than 1 year with acute tonsillitis prescribed antibacterials	0-20	13	72	12	75
ESAC4 B[a]	=4a receiving the recommended antibacterials: narrow spectrum penicillin	80-100	10	77	8	67
ESAC4 B[b]	=4a receiving the recommended antibacterials: any penicillin	80-100	13	100	12	100
ESAC4 C	=4a receiving quinolones	0-5	0	0	0	0
ESAC5 A	Percentage of patients older than 18 years with sinusitis, upper respiratory tract infection, or cold prescribed an antibacterial	0-20	2	17	5	9
ESAC5 B[b]	=5a receiving the recommended antibacterials: any penicillin	80-100	1	50	3	60
ESAC5 C	=5a receiving quinolones	0-5	0	0	0	0
ESAC7 A	Percentage of patients aged between 18 and 65 prescribed antibacterials	90-100	3	50	2	100

ESAC7 B[a]	=7a receiving the recommended antibacterials: broad spectrum penicillin or tetracycline	80-100	0	0	2	100
ESAC7 B[b]	=7a receiving the recommended antibacterials: any penicillin or tetracycline	80-100	0	0	2	100
ESAC7 C	=7a receiving quinolones	0-5	3	100	0	0

[a]Broad spectrum penicillin = J01CA: Extended spectrum penicillins; Narrow spectrum penicillin = J01CE, beta-lactamase sensitive penicillins

[b]Broad spectrum and narrow penicillins combined into one category applied to both J01CA and J01CE

UK:

		Acceptable Range (%)	PPAS 1(n)	PPAS 1 (%)	PPAS 4 (n)	PPAS 4 (%)
ESAC 1A	Percentage of patients aged between 18-75 with acute bronchitis/bronchiolitis prescribed antibacterials	0-30	28	78	21	88
ESAC1 B[a]	= 1a receiving the recommended antibacterials: broad spectrum penicillin or tetracycline	80-100	25	89	18	86
ESAC1 B[b]	=1a receiving the recommended antibacterials: any penicillin or tetracycline	80-100	26	93	19	91
ESAC1 C	= 1a receiving quinolones	0-5	0	0	0	0
ESAC2 A	Percentage of patients older than 1 year with acute upper respiratory infection, cold, or sinusitis, prescribed antibacterials	0-20	0	0	9	11
ESAC2 B[a]	=2a receiving the recommended antibacterials: narrow spectrum penicillin	80-100	NA	NA	0	0
ESAC2 B[b]	=2a receiving the recommended antibacterials: any penicillin	80-100	NA	NA	4	44
ESAC2 C	=2a receiving quinolones	0-5	NA	NA	0	0

ESAC4 A	Percentage of patients older than 1 year with acute tonsillitis prescribed antibacterials	0-20	53	77	18	95
ESAC4 B[a]	=4a receiving the recommended antibacterials: narrow spectrum penicillin	80-100	37	70	13	72
ESAC4 B[b]	=4a receiving the recommended antibacterials: any penicillin	80-100	44	83	14	78
ESAC4 C	=4a receiving quinolones	0-5	0	0	0	0
ESAC5 A	Percentage of patients older than 18 years with sinusitis, upper respiratory tract infection, or cold prescribed an antibacterial	0-20	0	0	7	16
ESAC5 B[b]	=5a receiving the recommended antibacterials: any penicillin	80-100	NA	NA	3	43
ESAC5 C	=5a receiving quinolones	0-5	NA	NA	0	0
ESAC7 A	Percentage of patients aged between 18 and 65 prescribed antibacterials	90-100	7	100	11	100
ESAC7 B[a]	=7a receiving the recommended antibacterials: broad spectrum penicillin or tetracycline	80-100	4	57	11	100
ESAC7 B[b]	=7a receiving the recommended antibacterials: any penicillin or tetracycline	80-100	4	57	11	100
ESAC7 C	=7a receiving quinolones	0-5	0	0	0	0

[a]Broad spectrum penicillin = J01CA: Extended spectrum penicillins; Narrow spectrum penicillin = J01CE, beta-lactamase sensitive penicillins

[b]Broad spectrum and narrow penicillins combined into one category applied to both J01CA and J01CE

Table S2. Denominator of the ESAC Quality Indicators by country for PPAS-1 (2020) and 4 (2022)

Country	Total (N) of patients aged 18-75 years with acute bronchitis /bronchiolitis		Total (N) of patients ≥1 year with acute upper respiratory infection		Total (N) of patients ≥1 year with acute tonsillitis		Total (N) of patients ≥18 years with acute/chronic sinusitis		Total (N) of patients aged 18-65 years with pneumonia	
	PPAS-1	PPAS-4	PPAS-1	PPAS-4	PPAS-1	PPAS-4	PPAS-1	PPAS-4	PPAS-1	PPAS-4
Belgium	29	0	68	65	45	3	52	42	11	1
Croatia	27	3	9	22	15	4	5	16	9	2
Denmark	51	9	29	34	38	15	20	20	27	6
Georgia	52	16	11	58	13	4	7	44	4	9
Germany	10	11	63	122	12	4	56	120	4	10
Greece	40	19	0	19	11	5	0	18	7	3
Ireland	35	6	49	55	26	12	40	30	11	3
Moldova	15	0	73	80	47	1	26	65	1	0
Netherlands	31	6	56	101	17	10	44	55	31	6
Poland	12	6	5	82	14	2	2	38	6	5
Romania	28	4	15	16	62	10	13	10	6	4
Spain	70	9	13	66	18	16	12	56	6	2
UK	36	24	45	79	69	19	24	43	7	11
Total	436	113	436	799	387	105	301	557	130	60

Text S1: Pre-defined questionnaire PPAS 1

Eligibility: register data on sequential patients who present with CA-ARTI with either:

a) acute cough (<28 days) as the predominant symptom, and/or the clinician judges the symptoms are due to a lower respiratory tract infection

b) acute sore throat (<14 days) or difficulty swallowing as the predominant symptoms, and/or the clinician judges the symptoms are due to pharyngitis or tonsillitis

Exclusion: Patients with only nasal, ear, rhinosinusitis symptoms

Site: AA00 Date:

Consultation at: □ office hours □ out-of-hours □ unknown

Patient characteristics

Age: .. years

Number of days with acute RTI symptoms before consulting: ... days

Any comorbidity present: □ yes □ no □ unknown

If yes: □ chronic respiratory condition (asthma, COPD, CF) □ diabetes □ cardiovascular disease

□ neoplasm □ chronic renal failure □ chronic neurological condition

□ other

Patient reported fever: □ yes □ no □ unknown

Temperature taken in clinic: □ yes □ no □ unknown

If yes, physician measured temperature: ...°C

Have you measured? Blood pressure: □ yes □ no □ unknown

O_2 saturation: □ yes □ no □ unknown

Respiratory rate: □ yes □ no □ unknown

Heart rate: □ yes □ no □ unknown

For adults: does the patient have a paid job? □ yes □ no □ unknown

For children: does the patient attend out-of-home care or school? □ yes □ no □ unknown

How many days has the patient missed work/out-of-home care/school? ... days □ unknown

Signs and symptoms

Ear pain: □ yes □ no □ unknown

Coryza: □ yes □ no □ unknown

Sore throat or difficulty swallowing: □ yes □ no □ unknown

If yes, tick all that apply: □ tonsillar exudate

□ swollen tonsils

□ tender cervical nodes
□ peritonsillar abscess

Cough: □ yes □ no □ unknown

If yes, tick all that apply: □ short of breath (dyspnea)
□ increased, or purulent sputum
□ abnormal auscultation
□ (pleuritic) chest pain
□ wheezing
□ tachypnea

General symptoms: □ yes □ no □ unknown

If yes, tick all that apply □ headache
□ altered mental status
□ palpitations
□ lethargy
□ reduced urine output

Overall illness severity (physician's impression): □ mild □ moderate □severe □unknown

Have you done/ordered additional diagnostic tests? □ yes □ no □ unknown

If yes:

□ Group A β-hemolytic Strep antigen	□ POC	□ LAB
□ CRP	□ POC	□ LAB
□ Procalcitonin	□ POC	□ LAB
□ RSV	□ POC	□ LAB
□ Influenza	□ POC	□ LAB
□ Total white blood cell count	□ POC	□ LAB
□ Legionella (urine)	□ POC	□ LAB
□ Pneumococci (urine)	□ POC	□ LAB
□ Multiplex PCR panel	□ POC	□ LAB

□ Chest X-ray
□ Other test: ……….

What was the suspected etiology: □ viral □ bacterial □ allergic □ not clear □ unknown

Initial working diagnosis □ acute pharyngitis □ acute tonsillitis □ laryngitis/laryngotracheitis (croup)
□ peritonsillar abscess □ influenza-like-illness
□ bronchiolitis □ acute bronchitis □ pneumonia
□ viral induced/associated wheezing □ exacerbation asthma or COPD
□ other: ……..

Medical history of antibiotic allergy/intolerance: □ yes □ no □ unknown

If yes, to which antibiotic? ………

Already treated with antibiotics in the past week? □ yes □ no □ unknown

Antibiotic prescribing: □ yes □ no □ unknown

For PC setting: If yes: □ immediate □ delayed

Which antibiotic class?

□ tetracycline (e.g. doxycycline, oxytetracycline)

□ narrow spectrum penicillin (e.g. pheneticillin, phenoxymethyl penicillin)

□ broad spectrum penicillin (e.g. amoxicillin)

□ co-amoxiclav (amoxicillin/clavulanate)

□ macrolide (e.g. erythromycin, clarithromycin, azithromycin)

□ quinolone (e.g. ciprofloxacin, moxifloxacin, levofloxacin)

□ cephalosporin (e.g. cephalexin)

□ other: ………….

Did the patient request an antibiotic prescription? □ yes □ no □ unknown

How confident were you about your (non-)prescribing decision?

□ very certain □ certain □ moderately □ uncertain □ very uncertain □ unknown

Have you provided? □ advice/prescribed days off school/work. If ticked, for how many days? ……..

□ advice for symptomatic treatment

□ other prescribed medication. If ticked: □ inhaled medication

□ antiviral medication

□ antihistamines

For PC setting: Did you refer the patient to hospital? □ yes □ no □ unknown

For hospital setting: Was patient discharged home □ yes □ no □ unknown

If no, patient admitted to: □ short stay assessment unit

□ inpatient ward

□ ICU/HDU

Text S2: Pre-defined questionnarie PPAS 4

Registration PPAS4 16NOV2021

Eligibility: register data on sequential patients who consult (F2F or phone/video) with either:

a) symptoms of a lower and/or upper respiratory tract infection (≤14 days)
b) another suspicion of COVID-19

Exclusion: Patients with only ear or allergic symptoms

ID: AA-X-000 Consultation date:

Consultation at/via: □ practice □ telephone □ video/skype □ home
□ urgent outpatient clinic □ ED

Consultation in this illness episode: □ first □ follow-up □ unknown

1 Has the patient been tested for COVID-19 in the past 2 weeks: □ yes □ no □ unknown
If yes: □ self-testing □ testing street □ GP/hospital
□ positive □ negative □ awaited

2 For F2F consultations, did you use personal protective equipment: □ yes □ no □ N/A
If yes: □ apron/body protection
□ face, nose/mouth protection
□ safety glasses
□ gloves

3 Patient vaccinated for

COVID-19: □ yes □ no □ unknown
If yes: □ 1 dose □ 2 doses □ 3 doses
which vaccine(s): ………………..

Influenza: □ yes □ no □ unknown

Pneumococci: □ yes □ no □ unknown
If yes: □ PCV7 □ PCV10 □ PCV13 □ PPV23 □ unknown

Patient characteristics

4 Sex: □ male □ female

5 Age: …. months (0-11 months) …. years (≥1 year of age)

6 Number of days with acute RTI symptoms before this consultation: ... days

7 Comorbidity present: □ yes □ no

If yes: □ chronic respiratory condition (asthma, COPD, CF) □ diabetes □ cardiovascular disease

□ obesity □ other

8 Patient/parent reported fever: □ yes □ no

9 Have you measured? Temperature: □ yes □ no If yes: …….°C

O_2 saturation: □ yes □ no If yes: ……. %

Respiratory rate: □ yes □ no

Heart rate: □ yes □ no

Signs and symptoms (either reason for consulting or part of consultation)

10 Rhinitis: □ yes □ no □ unknown

11 Sore throat or difficulty swallowing: □ yes □ no □ unknown

If yes, tick all that apply: □ tonsillar exudate

□ swollen tonsils

□ tender cervical nodes

□ peritonsillar abscess □ none of the above □ not examined

12 Cough: □ yes □ no □ unknown

If yes, tick all that apply: □ short of breath (dyspnea)

□ increased, or purulent sputum

□ abnormal auscultation

□ wheezing

□ breathing fast (tachypnea) □ none of the above □ not examined

13 General symptoms: □ yes □ no □ unknown

If yes, tick all that apply □ headache

□ muscle ache

□ fatigue/extremely low energy

□ diarrhea

□ loss of taste/smell □ none of the above □ not examined

14 Overall illness severity (GP's impression): □ mild □ moderate □ severe

15 Have you done/ordered additional diagnostic tests? □ yes □ no

If yes: □ Group A β-hemolytic Strep antigen □ POC □ LAB

If POC: pos / neg

□ CRP □ POC □ LAB

If POC: value ……..

□ Influenza □ POC □ LAB

If POC: pos / neg

□ COVID-19 test □ POC □ Lab

If POC: antigen / PCR pos / neg

□ Total white blood cell count □ POC □ LAB

□ Chest X-ray

□ Other test, specify: …………..

16 What is the suspected etiology: □ viral (other than SARS-CoV-2) □ SARS-CoV-2 □ bacterial □ not clear

17 How certain are you about this suspected etiology:

□ very certain □ certain □ moderately □ uncertain □ very uncertain

18 Working diagnosis: □ acute pharyngitis □ acute tonsillitis □ laryngitis/laryngotracheitis (croup)

□ peritonsillar abscess □ bronchiolitis □ acute bronchitis □ CA pneumonia □ infectious wheeze □ exacerbation of chronic respiratory condition

□ Influenza □ COVID-19

□ upper RTI / common cold / sinusitis □ other, specify: ……….

19 Have you? □ prescribed medication, if ticked: □ inhaled medication

□ antibiotic, if ticked, which one: ……………………..

□ antiviral medication, if ticked, which one: ….........

□ antihistamines

□ other

□ advised/prescribed days off work and/or school

□ advised home isolation/quarantine

□ advised symptomatic treatment

□ scheduled a follow-up visit/call

□ advised on preventive measures for patient and/or family members

□ none of the above

20 How confident are you that the medication you prescribed will benefit this patient:

□ very confident □ confident □ moderately □ unconfident □ very unconfident □ NA

21 Did you refer the patient to hospital? □ yes □ no
